# Active Living: development and quasi-experimental evaluation of a school-centered physical activity intervention for primary school children

**DOI:** 10.1186/s12889-015-2633-1

**Published:** 2015-12-29

**Authors:** Dave H. H. Van Kann, M. W. J. Jansen, S. I. de Vries, N. K. de Vries, S. P. J. Kremers

**Affiliations:** Department of Health Promotion, School of Public Health and Primary Care (CAPHRI), Maastricht University, P.O. Box 616, 6200 MD Maastricht, The Netherlands; Academic Collaborative Center for Public Health Limburg, Public Health Services, Geleen, The Netherlands; Department of Health Services Research, School of Public Health and Primary Care (CAPHRI), Maastricht University, P.O. Box 616, 6200 MD Maastricht, The Netherlands; TNO, Expertise Center LifeStyle, Leiden, The Netherlands; The Hague University of Applied Sciences, Research group Healthy Lifestyle in a Supporting Environment, P.O. Box 13336, 2501 EH The Hague, The Netherlands; Department of Health Promotion, Nutrition and Translational Research Institute Maastricht (NUTRIM), Maastricht University, P.O. Box 616, 6200 MD Maastricht, The Netherlands

**Keywords:** Longitudinal evaluation, Quasi-experimental design, Multicomponent interventions, Physical activity, Accelerometry, Primary school environment, Children

## Abstract

**Background:**

The worldwide increase in the rates of childhood overweight and physical inactivity requires successful prevention and intervention programs for children. The aim of the Active Living project is to increase physical activity and decrease sedentary behavior of Dutch primary school children by developing and implementing tailored, multicomponent interventions at and around schools.

**Methods/design:**

In this project, school-centered interventions have been developed at 10 schools in the south of the Netherlands, using a combined top-down and bottom-up approach in which a research unit and a practice unit continuously interact. The interventions consist of a combination of physical and social interventions tailored to local needs of intervention schools. The process and short- and long-term effectiveness of the interventions will be evaluated using a quasi-experimental study design in which 10 intervention schools are matched with 10 control schools. Baseline and follow-up measurements (after 12 and 24 months) have been conducted in grades 6 and 7 and included accelerometry, GPS, and questionnaires. Primary outcome of the Active Living study is the change in physical activity levels, i.e. sedentary behavior (SB), light physical activity (LPA), moderate-to-vigorous physical activity (MVPA), and counts-per-minute (CPM). Multilevel regression analyses will be used to assess the effectiveness of isolated and combined physical and social interventions on children’s PA levels.

**Discussion:**

The current intervention study is unique in its combined approach of physical and social environmental PA interventions both at school(yard)s as well as in the local neighborhood around the schools. The strength of the study lies in the quasi-experimental design including objective measurement techniques, i.e. accelerometry and GPS, combined with more subjective techniques, i.e. questionnaires, implementation logbooks, and neighborhood observations.

**Trial registration:**

Current Controlled Trials ISRCTN25497687 (registration date 21/10/2015), METC 12-4-077, Project number 200130003

## Background

All over the world, the prevalence of childhood obesity has increased substantially within one generation [1]. As in most developed countries [2], the proportion of overweight and obese children in the Netherlands has tripled between 1980 and 2009, to 16 % [3]. The prevalence of overweight is higher among children with a low socio-economic (SES) background [4, 5]. Overweight in children is likely to track into adulthood [6, 7], adding to the need for childhood overweight and obesity prevention. The increase in overweight is particularly caused by changed lifestyle behaviors, such as decreased physical activity (PA) and increased sedentary behavior (SB) [8, 9], which are nowadays considered two independent risk factors for health, rather than each other’s counterparts [10, 11]. Physical inactivity and sedentary behavior are associated with a wide variety of chronic diseases [12], psychosocial problems [13], and impaired cognitive functioning [14]. Decreased PA and increased SB are likely to be a result of changed environmental factors, such as decreased road safety [15, 16] and increased availability of ‘screen-based devices’ (computers, televisions, tablets etc.) [17].

To date, there have been many interventions that aim to increase children’s physical activity levels, especially in the school setting [18, 19]. Schools are suitable settings for health promotion activities in children in view of their substantial reach [20], which greatly affects the potential impact of an intervention. In addition, the educational system is a learning environment, in which developing a healthy lifestyle could be considered an important objective. Despite these potentials, however, schools are not able to solve the problem of physical inactivity by themselves [21, 22]. Nonetheless, most school-based PA interventions focus solely on the school setting [23–26], whereas it could be argued that school is only one level of influence [27] and interactions with different ecological levels (e.g. home environment or local neighborhood) are more likely to change health behaviors [28].

**Fig. 1:**
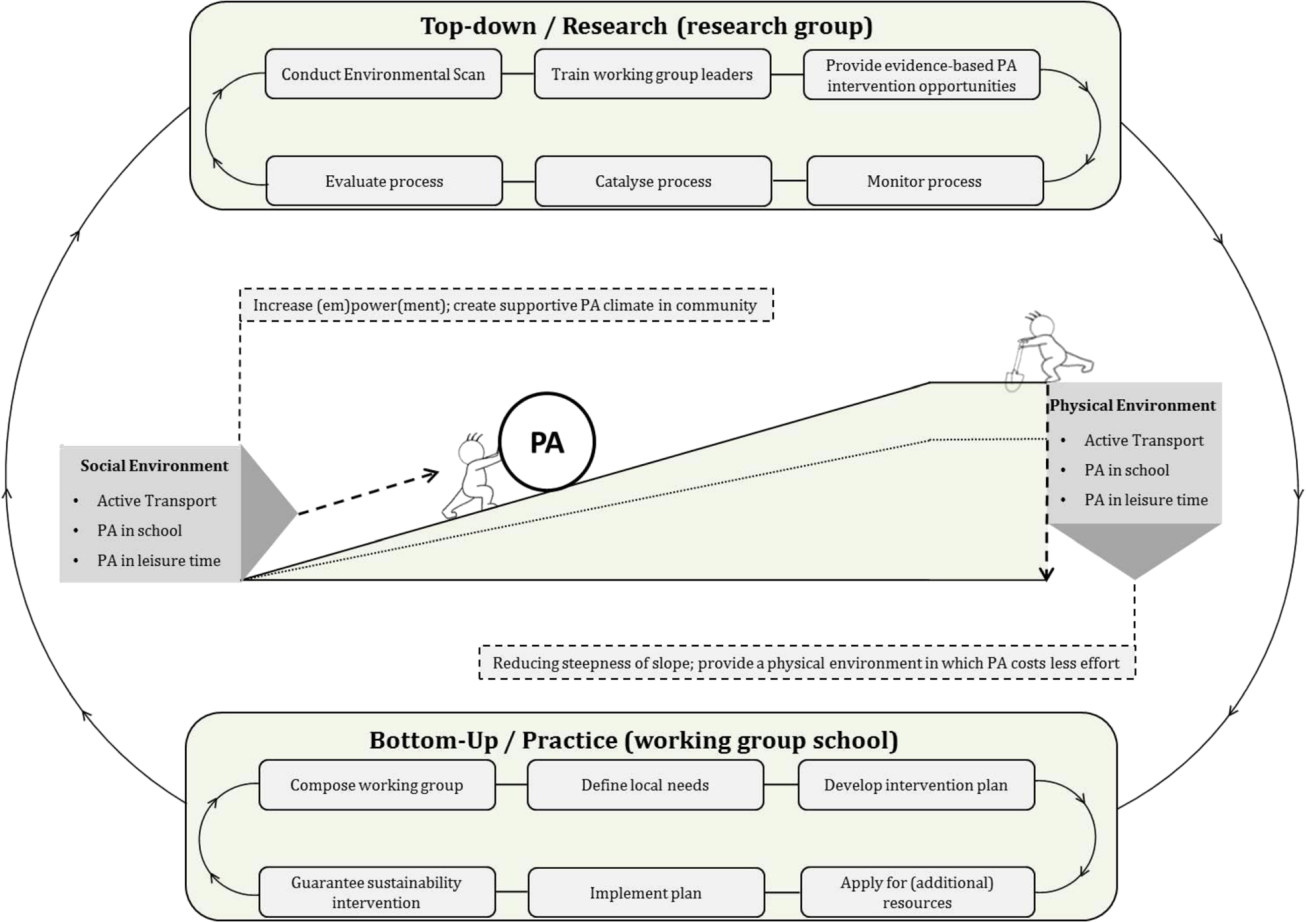
Active Living development and implementation loop

The current project focuses on promoting children’s PA levels (i.e. active school transportation, PA while attending school, and leisure time PA) in the school setting in a broad sense, including local neighborhoods and parts of the home environment. The Active Living project intends to create PA-friendly school environments by creating a supportive physical environment (e.g. safe routes to school and active schoolyards) accompanied by a supportive social environment (e.g. parental support to walk to school and facilitative teacher practices in schoolyards) (Fig. 1). The interaction between interventions in both types of environments [29] is hypothesized to affect PA and SB favorably [28].

The aim of the Active Living project is to develop and implement tailored, multicomponent school-centered PA interventions and test their short- and long-term effectiveness regarding the PA levels of children aged 8–12 years living in deprived areas.

## Method

### Setting and study design

**Fig. 2:**
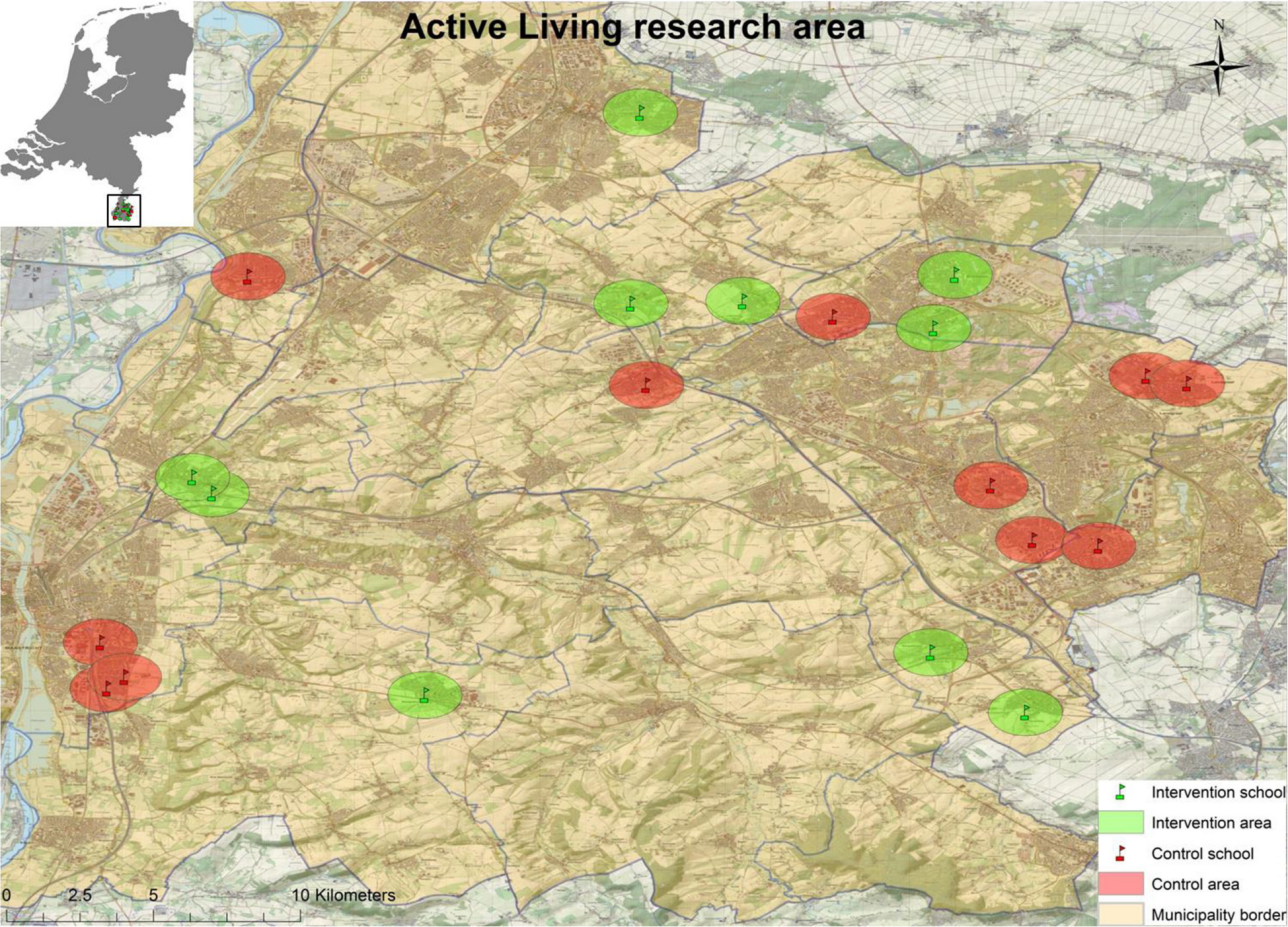
Active Living research area – Southern-Limburg region, The Netherlands

The current study uses a quasi-experimental design with 10 intervention schools and 10 matched control schools from the Southern-Limburg area in the Netherlands (Fig. 2). It uses a combination of selective and universal prevention. Selective prevention is reflected by the focus on schools in deprived areas [30], with larger proportions of children from lower SES backgrounds. The project is, however, not only designed for low-SES children, but focuses on all children attending the schools in the deprived areas. ‘Active Living’ is funded by the Netherlands Organization for Health Research and Development (ZonMW), Project Number 200130003 (ISRCTN25497687). Ethical approval was obtained from the research ethics committee of the Maastricht University Medical Centre (reference number METC 12-4-077).

### Power calculation

This project aims to increase children’s physical activity levels. More specifically, its target is an increase in the proportion of children meeting the Dutch guideline for physical activity (60 min MVPA daily) from 22 % to 42 % after the project [31, 32]. Interclass variations within the school (0.3) and variations among the schools (0.7) were taken into account in the power calculation. In total, we calculated that 704 children would be needed to obtain sufficient power. In order to achieve this we needed to include 16 schools (8 intervention and 8 control schools). Due to potential differences in class sizes and risk of drop-out during the study, the number of schools was increased by 25 % to 20 schools (10 intervention and 10 control schools) and a total of 880 children.

### Recruitment of schools and participants

**Fig. 3:**
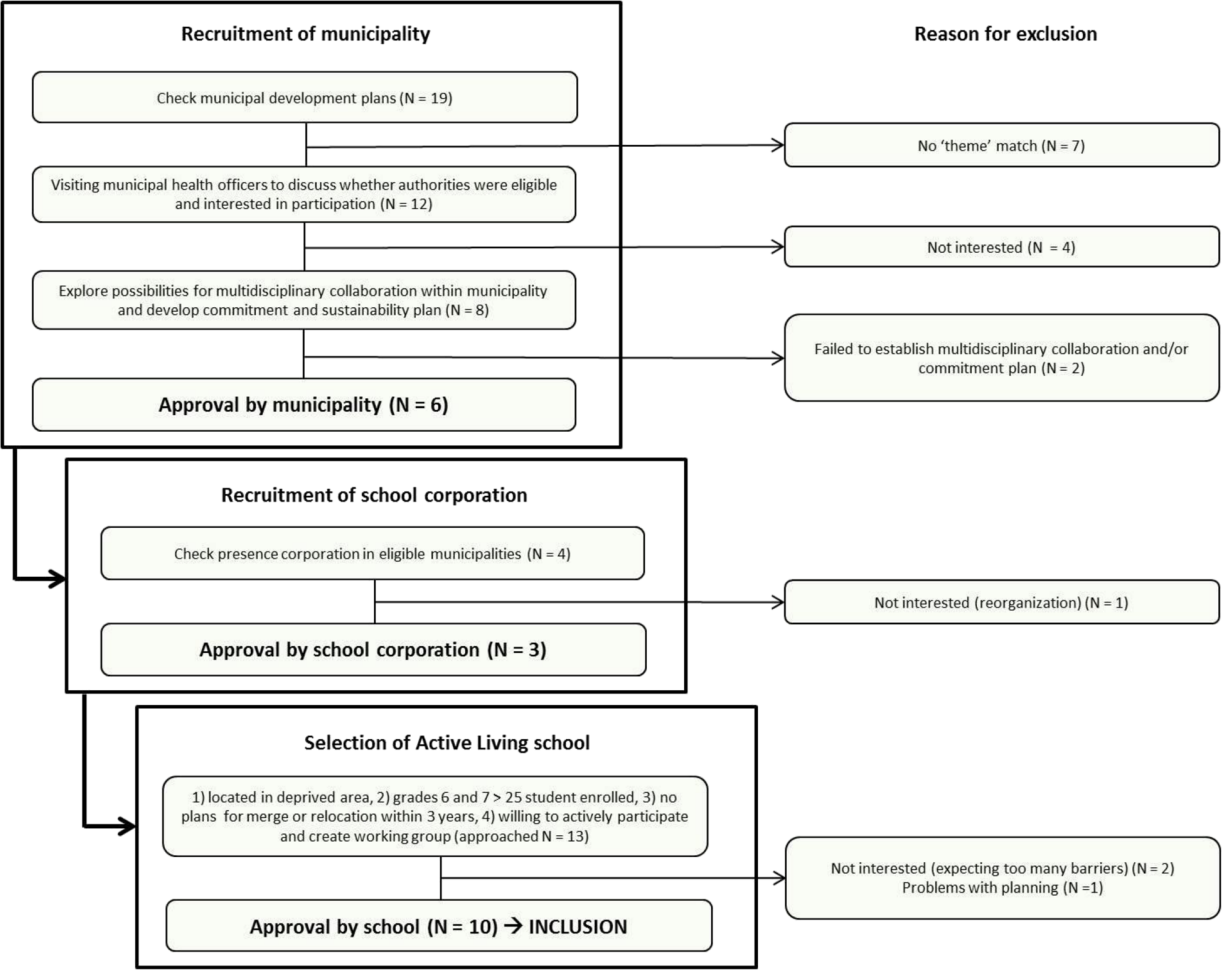
Recruitment of Active Living intervention schools

The Active Living project targets 6^th^-to-8^th^ grade children (8 to 12 years old) attending primary schools situated in deprived areas in the Southern-Limburg region. In November 2011, municipal development plans of all 19 Southern-Limburg municipalities were checked to see if they contained formal references to the themes of ‘youth’ and ‘overweight prevention’. In total, 12 out of 19 municipalities had formulated targets either for youth or overweight prevention or both. Municipal health officers were visited and informed about the project and conditions for participation. This resulted in six municipalities (31.6 %) wanting to become involved. In these municipalities, four school corporations were identified, three of which agreed to recruit schools falling under their responsibility. To participate in the Active Living project, schools were checked for eligibility according to predefined inclusion criteria: (1) located in a deprived area; (2) at least 25 students enrolled in grades 6 and 7; (3) no plans to merge with another school or plans to relocate in the upcoming 3 years; and (4) willing to actively participate and to form an ‘Active Living’ working group at school. Within the municipalities and school corporations that consented, 37 primary schools were identified in deprived areas. Municipalities and corporations were asked to recommend schools that were most eligible to participate from their perspective. We visited 13 eligible schools and informed them about the project and conditions for participation; 10 schools (76.9 %) agreed to participate (Fig. 3). Each of these schools was matched to a control school, taking into account the level of neighborhood deprivation and the level of urbanization (urban vs rural). Ideally, the control school was located in a non-participating municipality, to prevent potential contamination by municipal policy influences. After 10 matching control schools had been recruited, one control school unexpectedly had to relocate to a temporary school building and was thus replaced by an additional control school. In all, therefore, we included 11 control schools, 10 of which were matched to an intervention school. Control schools were only visited during measurements, and no project activities were planned during the intervention period, which comprised two academic years. Control schools were offered Public Health Services (PHS) support to implement effective elements of the project after the end of the effectiveness study.

**Fig. 4:**
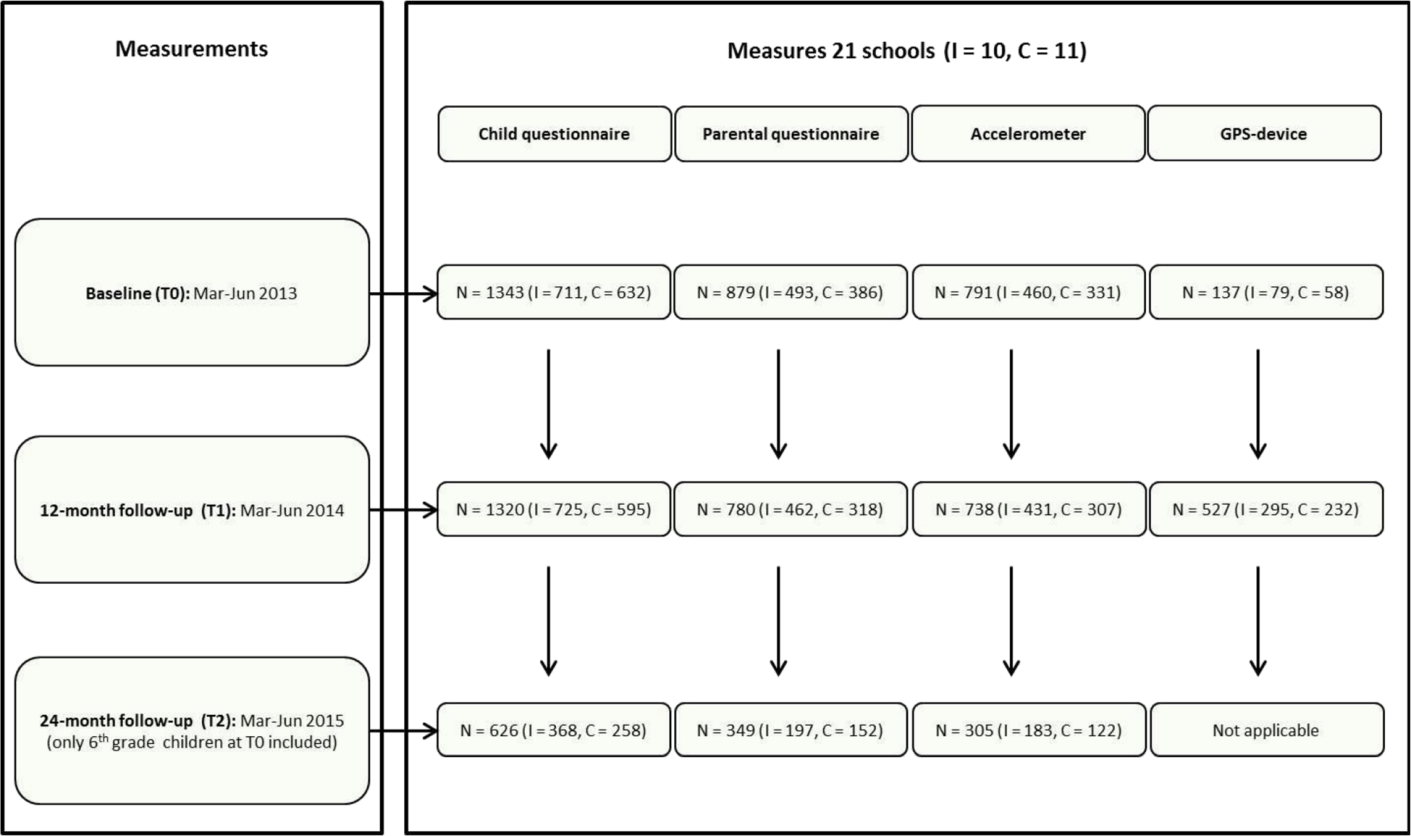
Overview of participants to the Active Living project

Before data collection, all participating schools were visited to inform children about the study. Children were able to express their interest in participating in the effect study of the Active Living project. Those children who were interested were provided with written information for their parents. Written informed consent was requested from parents for wearing measurement equipment before and during the project. School principals consented to administer the child questionnaire in the classroom and to ask the parents to fill out the parental questionnaire. After permission was received from the parents to include a child in the study, the child could still refuse further participation in any part of the study without giving any reason. At baseline, parents of 815 children (61.6 %) consented for them to wear the measuring equipment. Figure 4 presents an overview of the participants of the Active Living project.

### Data collection

The baseline measurement (T0) of the Active Living Study was performed between September and December 2012. The first follow-up measurement (T1) was performed 6 months later, between March and June 2013, and a second follow-up (T2) after 18 months, between March and June 2014. To prevent potential seasonal effects, data in each pair of schools (the intervention school and the ‘matched’ control school) were collected on the exact same dates. Grades 6 and 7 of participating schools were visited by two researchers and a PHS employee at all measurement moments. During classroom visits lasting about 1 h, children were instructed about the procedure of the measurement, were equipped with measurement devices (accelerometer and/or GPS device, see *Measures*), and individually filled out a questionnaire, which was immediately collected by the researchers. Afterwards, children received a parental questionnaire and were requested to ask their parents to fill it out and return it to school in a supplied envelope. A research assistant visited the schools one week afterwards to collect the measurement equipment and parental questionnaires.

Unexpectedly, the intervention implementation was delayed by at least 6 months as a result of reorganizations at our main executive partner (PHS). As a result, we revised the measurement design of our effectiveness study. The initial T0 measurement was redefined as a pilot and feasibility test (T-1). The measurement in the spring of 2013 was redefined as the baseline measurement (T0) while the original 18-month measurement became the first follow-up in the effectiveness trial (T1 = 12 months). As a result of this, we performed an additional follow-up measurement in the period of March till June 2015 (T2 = 24 months). This will allow us to assess the sustainability of potential effects of the Active Living project on the PA and SB behavior of the children who were in the 6^th^ grade at baseline. Children attending 7^th^ grade at the start of the project made the transition from primary to secondary school during the summer of 2014, and were therefore no longer eligible to participate in the additional 24-month measurement (T2).

The revised measurement design led to a loss of power in the study of long-term effects. On the other hand, it increased the comparability between the baseline (T0) and follow-up measurements (T1 and T2), since all measurements were now performed in spring (while T-1 was performed in the fall). In addition, a potential novelty effect of wearing measurement devices was prevented by excluding T-1 from the effectiveness trial.

### Measures

#### Accelerometry and GPS

PA was measured by waist-worn accelerometers (Actigraph GT3X+, 30Hz). Children were instructed to wear a belt, with the attached accelerometer on their right hip, for at least 5 consecutive days, including a weekend. They were asked to wear the device all day long during waking hours and only remove the belt when performing (substantial) water-involving activities, such as swimming and showering.

At T-1 and at T0, a random selection of children were also asked to wear a GPS device (QStarz BT1000-XT), which was added to their accelerometer belt. We opted for this random approach because of the limited availability of GPS devices. Children additionally wearing a GPS device followed a similar protocol as described for accelerometers, but were asked to charge the device overnight using a supplied adapter. At T1 our GPS capacity was greatly increased, allowing us to ask all children to wear a GPS device in addition to their accelerometer. T2 was designed as an accelerometer-only measurement, to prevent high drop-out rates, as the GPS device was thought to raise the burden of participation for the children.

#### Child questionnaire

The child questionnaire included items that can be classified into six main themes (Table 1): demographics; PA and SB; neighborhood perceptions; rules and practices at home; PA preferences, and impulsivity [33–36]. The questionnaire was pre-tested with children of the target age group to ensure the questions were clear and understandable. The questionnaire was designed to be completed individually by children within approximately 15 min. At T1, some process evaluation items were added to the questionnaire.

**Table 1:** Overview of concepts in the child questionnaire

Theme/concept	N items	Example of items
Demographics	9	Gender; Date of birth; Home address
Transportation options	3	How did you come to school this morning? (MC-9)
PA & SB	12	On how many days per week do you play outdoors? (Open) + duration question
Neighborhood perceptions	7	There is heavy traffic in our local streets (Lik-4)
Rules and practices at home	4	At home we have rules about using the computer or watching TV (Lik-4)
PA preferences	28	Visualized comparisons, e.g. outdoor play vs. reading
Impulsivity	13	I often rush into new things (Lik-5)
Process	4	On a scale from 1–10, how much fun do you think Active Living was to participate in? (SC)

*MC-9* Multiple Choice (9 options), *Lik-X* X-points Likert Scale, *SC* Scale

#### Parental questionnaire

The parental questionnaire included nine main topics (Table 2): demographics; school transportation choices; neighborhood perceptions; parental PA practices; child’s outdoor play; parental active transportation routines (PATRns); rules; biking behavior, and self-reported height and weight of the child, mother, and father [37, 38]. The questionnaire was designed to be completed in 10 min, which was believed to reduce the ‘time barrier’ to filling out the questionnaire.

**Table 2:** Overview of concepts in the parental questionnaire

Theme/concept	N items	Example of items
Demographics	11	Relation to child; Level of education
School transportation options	3	Who accompanies your child during transportation to school? (MC-6)
Neighborhood perceptions	8	Social safety is a concern in our local neighborhood (Lik-4)
Parental PA practices		
Modeling	6	How often do you use your own behavior to encourage your child’s PA? (Lik-5)
Facilitation	2	How often do you bring your child to a location where he/she is able to do sports or be physically active? (Lik-5)
Social Support	8	How often do you stimulate your child to be physically active? (Lik-5)
Child’s outdoor play	10	If my child does not play outdoors, it is because…. there is no playground in the local neighborhood (Lik-3)
Parental Active Transportation Routines (PATRns)	4	If I have to go somewhere nearby, I am always inclined to take the bike or to go on foot (Lik-5)
Rules	4	How often do you restrict the time your child can use the computer? (Lik-5)
Biking behavior	4	How often do you cycle yourself? (Lik-5)
Anthropometry	6	What is your height and weight? (Open)

*MC-6* Multiple Choice (6 options), *Lik-X* X-points Likert Scale

#### Environmental data

In August 2012, prior to the start of the project at the schools, an environmental scan of all school environments was conducted using the SPACE checklist [39]. This is an adapted version of the Neighborhood Environment Walkability Scale (NEWS) [40], specifically adapted to and validated for the Dutch context. A school environment was defined as an 800 m crow-fly buffer around every primary school. In 2012, Dutch primary school children lived at an average distance of 600 m from their nearest primary school [41], so the 800 m crow-fly buffer seemed appropriate to include both the school and home environment of the primary school children and their route to school. Two trained researchers conducted the environmental scan by walking through the neighborhood. Each scan took about 3–4 h to complete. Environmental attributes that were audited were classified into: ‘Schoolyard characteristics’ (e.g. play equipment, green spaces, benches), ‘Residential buildings’ (e.g. numbers of apartment blocks), ‘Sports facilities’ (e.g. presence of sports fields), ‘Playgrounds’ (e.g. numbers of playgrounds, equipment at playgrounds, accessibility), ‘Parks’ (e.g. presence of park, play equipment in park, accessibility), ‘Green spaces and water’ (e.g. quantity, accessibility), ‘Street networks’ (e.g. presence of sidewalks, quality of sidewalks), ‘Traffic safety in school vicinity’ (e.g. presence of busy roads, heavy traffic), ‘Street hygiene’ (e.g. presence of litter, dog waste), ‘Social safety’ (presence of hang-outs, dark places), and ‘Cyclability and walkability of neighborhood’ (general impression of neighborhood). All study areas were revisited after T2 (12 months) to check for environmental changes. In addition, contextual information on the school environment (e.g. residential density) was ‘enriched/supplemented’ using the Geographic Information System (GIS) (ArcGIS, version 10.2, Top10NL). Furthermore, weather conditions, i.e. temperature, precipitation and hours of sunshine, were obtained for every hour of a measurement day from the Royal Netherlands Meteorological Institute (KNMI).

### Procedures of intervention development and implementation

A combined top-down and bottom-up approach was used to develop and implement tailored, multicomponent school-centered PA interventions (Fig. 1). The ‘Active Living’ project involved a project team that was composed of a research unit using the top-down approach and a practice unit using the bottom-up approach. Both units were based in the Academic Collaborative Center for Public Health, which facilitated continuous information exchange, leading to ongoing adjustments to the project while in progress. Subsequently, researchers conducted the environmental scans and trained three PHS employees in ‘(physical/social) environmental thinking’, and provided evidence-based PA intervention opportunities. After the start of the project the research unit was involved in monitoring, catalyzing, and evaluating the process, and made adjustments when needed. The trained PHS employees composed a working group at each intervention school consisting of at least the PHS employee as chair, and representatives of the school, parents, and municipal authorities (ideally multidisciplinary, i.e. municipal officials from different disciplines/departments) and was complemented by other stakeholders when possible and necessary. The working groups started with a small budget of 2000 euros each for a period of two school years. Informed by the environmental scan, this working group defined local needs. Based on the needs assessment, an intervention and sustainability plan was formulated, which was assessed by the project team before equipment or training facilities for the interventions were funded. If plans exceeded the budget, additional funding resources were applied for. After (financial) approval by the project team, intervention plans were implemented and monitored for their impact. Meanwhile the bottom-up loop was repeated for additional PA interventions.

### Statistical analyses

PA as measured with accelerometers will be described as activity counts-per-minute (CPM) and by activity levels, i.e. sedentary behavior (SB), light physical activity (LPA), and moderate-to-vigorous physical activity (MVPA). Activity levels will be classified using Evenson’s cut-off values [41]. The primary outcome variables for the effectiveness study of Active Living will be the change in CPM (Δ CPM) and the change in time spent in SB, LPA, and MVPA (Δ time spent in SB, LPA and MVPA, respectively) between T1 and the extended baseline measurement (T0). Differences between T2 and T1 and the extended baseline measurement (T0) will be analyzed to assess the sustainability of the Active Living intervention effects. Multivariate multilevel regression analyses will be used to adjust for the nested structure of data in schools. The regression analyses will also be adjusted for age, gender, ethnicity, and weather conditions. Intervention effects of multicomponent physical and social interventions will be studied, as well as their effectiveness under specific conditions, i.e. supportive school environments or home environments. The influence of moderators, such as child characteristics or parental practices, on the intervention effects on children’s PA will be studied by including interaction terms of potential moderators in the models. In case of significant moderation, stratified analyses will be used to elucidate conditional factors.

Moreover, we intend to combine objectively (accelerometry and GPS) and subjectively (questionnaires) assessed measures to study the influence of environmental features (observed and perceived), children’s characteristics (PA preferences and impulsivity), and parental practices on children’s PA. Additional research questions have been formulated, such as ‘Do PA preferences predict physical activity?’ or ‘What is the influence of parental PA practices on children’s PA levels?’. The GPS data will allow us to zoom in on certain potentially important environments, such as schoolyards, playgrounds, or routes to school. Due to the limited number of GPS devices (at baseline), these location-specific data will not be part of the effectiveness study, but will be used for additional research questions, such as ‘What neighborhood characteristics influence the use of active transportation?’.

## Discussion

The current paper has discussed the development and quasi-experimental evaluation of a school-centered PA intervention. In addition, we have introduced the plan of analysis to investigate the effectiveness of multilevel, multicomponent PA interventions on children’s PA, which were developed and implemented using a combined top-down and bottom-up approach. We have also presented an overview of the recruitment procedures and measures, and elaborated on the research questions we will try to answer from our data, in addition to the main study objective.

The Active Living project involved developing and implementing a set of tailored physical and social environmental PA interventions. Every school had a different starting point for this study, and local needs varied greatly, both in focus (active transportation, PA at school, or PA in leisure time) and in content. The effectiveness of the Active Living project will be evaluated as a multilevel (individual, school, and/or neighborhood) multicomponent intervention. In this perspective, we focus on studying the effectiveness of changing the PA-friendliness of the whole school environment to enhance PA, rather than evaluating specific types of interventions. We will report on types of interventions implemented as part of Active Living to create a measure of the magnitude of the total set of physical and social interventions. We believe that studying the tailored development and implementation of a set of interventions in a combined top-down and bottom-up approach is one of the strengths of the Active Living project. Other strengths of the study are our measurements, including both objective assessment methods (accelerometry, GPS, and GIS in combination with environmental scans) and subjective methods (questionnaires among both children and their parents). The objective measurement techniques have already been tested in previous studies using similar target populations [42–44] and enable us to study children’s actual physical activity behavior in a broader context, compared to previously used observation tools, such as SOPLAY and OSRAC-H [45, 46].

The Active Living study has a quasi-experimental design. RCTs are generally considered to be the gold standard for testing the effectiveness of an intervention [18]. For practical reasons, however, an RCT design seems less appropriate for field studies in which environmental changes are conducted in a wider, less controlled study area, as is the case in the Active Living project. Each of our study areas covers an environmental surface of 2 km^2^, certain parts of which will not be visited by all participants. Therefore, the exposure to interventions is more difficult to define than in studies focusing on one particular controlled area, such as a schoolyard. Besides, participants are well aware of whether they belong to an intervention school/area, since physical changes are directly visible to participants. Blinding is therefore practically impossible. Furthermore, adaptations in the public municipal domain have to be approved (and co-designed and co-funded) by local authorities and they may be key to the successful implementation of a participatory intervention strategy. Nonetheless, the quasi-experimental research design follows many of the RCT assumptions, and allows us to correct for potential confounding factors, such as socio-cultural and political changes to the environment and weather conditions. Moreover, the quasi-experimental study design seems to be suitable for testing the effectiveness of interventions in daily practice, and therefore may facilitate the implementation and continuity of interventions.

## Conclusion

The Active Living project contributes to the knowledge about the relationship between environmental interventions and physical activity. Active Living uses a participatory intervention development strategy, and multicomponent physical and social environmental PA interventions will be evaluated for their effects. The outcomes of the Active Living project will guide future projects to design or redesign schools, schoolyards, and their local environment.

## Abbreviations

CPM: Counts-per-minute
GIS: Geographic Information System
GPS: Global Positioning System
LPA: Light physical activity
MVPA: Moderate-to-vigorous physical activity
OSRAC-H: Observational System for Recording Physical Activity in Children–Home
PA: Physical activity
PHS: Public Health Service
RCT: Randomized Clinical Trial
SB: Sedentary behavior
SOPLAY: System for Observing Play and Leisure Activity in Youth
